# ECKN: An Integrated Approach for Position Estimation, Packet Routing, and Sleep Scheduling in Wireless Sensor Networks

**DOI:** 10.3390/s23136133

**Published:** 2023-07-04

**Authors:** Mauricio Bertanha, Richard W. Pazzi, Khalil El-Khatib

**Affiliations:** Faculty of Business and IT, Ontario Tech University, 2000 Simcoe St N., Oshawa, ON L1G 0C5, Canada; mauricio.bertanha@ontariotechu.ca (M.B.); khalil.el-khatib@ontariotechu.ca (K.E.-K.)

**Keywords:** wireless sensor networks, position estimation, greedy geographic forwarding, packet routing, sleep scheduling, duty cycling, mobile sink, performance evaluation, data dissemination protocol

## Abstract

Network lifetime and localization are critical design factors for a number of wireless sensor network (WSN) applications. These networks may be randomly deployed and left unattended for prolonged periods of time. This means that node localization is performed after network deployment, and there is a need to develop mechanisms to extend the network lifetime since sensor nodes are usually constrained battery-powered devices, and replacing them can be costly or sometimes impossible, e.g., in hostile environments. To this end, this work proposes the energy-aware connected k-neighborhood (ECKN): a joint position estimation, packet routing, and sleep scheduling mechanism. To the best of our knowledge, there is a lack of such integrated solutions to WSNs. The proposed localization algorithm performs trilateration using the positions of a mobile sink and already-localized neighbor nodes in order to estimate the positions of sensor nodes. A routing protocol is also introduced, and it is based on the well-known greedy geographic forwarding (GGF). Similarly to GGF, the proposed protocol takes into consideration the positions of neighbors to decide the best forwarding node. However, it also considers node residual energy in order to guarantee the forwarding node will deliver the packet. A sleep scheduler is also introduced in order to extend the network lifetime. It is based on the connected k-neighborhood (CKN), which aids in the decision of which nodes switch to sleep mode while keeping the network connected. An extensive set of performance evaluation experiments was conducted and results show that ECKN not only extends the network lifetime and localizes nodes, but it does so while sustaining the acceptable packet delivery ratio and reducing network overhead.

## 1. Introduction

A wireless sensor network (WSN) consists of a large number of low-cost devices, or sensor nodes, which have wireless communication capabilities. These networks enable various applications in domains such as health, military, and security [1]. Sensor nodes in these networks can be used to track, monitor, or control a large area without the need for fixed infrastructure [2,3,4,5]. WSNs can be used in a wide variety of applications, including monitoring, safety, and border control, just to name a few. A WSN with mobile sinks can be used in precision agriculture, for instance, where a drone or vehicle moves around the field to collect information from sensor nodes and, in our solution, to provide points of reference for sensor nodes to perform trilateration and localize themselves. Sensor nodes are usually deployed randomly, therefore knowing their positions is crucial: forest fire data, for instance, are useless without clear coordinates for where the data are coming from. In addition, extending the network lifetime is crucial in such hostile environments. Sensor nodes along a routing path that deplete their energy resources fast may render the entire network inoperational. The proposed ECKN takes into consideration the energy remaining in sensor nodes to make decisions on duty cycles.

Coverage and connectivity are two performance metrics that are mainly studied in WSNs, as described by Li et al. [6]. Coverage refers to the surveillance map of the area, and in this metric, nodes placed in a region cooperate with each other in order to maintain good coverage quality. Some benchmarks are used to quantify coverage quality in terms of granularity. At the highest granularity, every point on the map should be covered by at least one sensor. At the medium granularity, every path crossing the network should be covered. Finally, at the lowest granularity, the map may not be entirely covered by nodes, and the network may not necessarily be connected.

Connectivity, on the other hand, refers to message retrieval and delivery in the network. The connectivity of a node to other nodes in the network can be considered in two dimensions related to energy consumption. In the temporal domain, nodes switch between active and sleep states. In the spatial domain, nodes have multiple energy levels that vary their communication ranges.

In WSN applications, being location-aware is an important factor in fundamental tasks such as packet routing, event mapping, and energy savings. However, due to the deployment of a large number of sensor nodes, manual placement of these devices is often unfeasible and costly. Consequently, sensor nodes usually acquire their positions through location estimation. One of the most common localization methods is the global positioning system (GPS) [7]. However, for small, inexpensive, low-power devices that are left unattended for long periods of time, the use of GPS modules on all sensor nodes is unfeasible due to size, form factor, cost, and power constraints [8].

**Figure 1:**
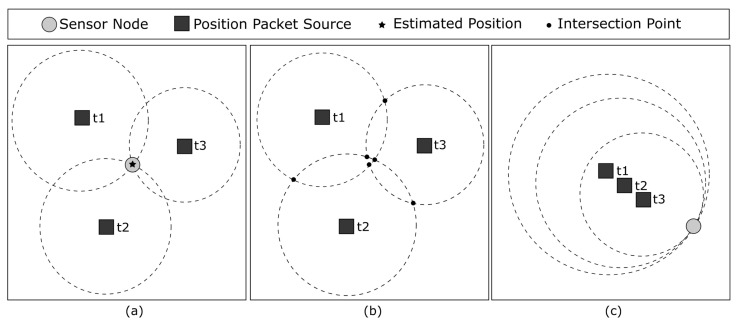
(**a**) Position of sensor node defined by trilateration. (**b**) GPS error added to sink packets, the intersection between the packets is not a single point. (**c**) Poor position packet samples to perform trilateration.

In this paper, we present an integrated node localization, sleep scheduling, and packet routing algorithm. The proposed mechanism estimates the positions of sensor nodes by exchanging position packets between a mobile sink and the sensor nodes. As the mobile sink is more powerful, it houses a GPS module and shares its position within the network. When a sensor node receives a position packet, it uses a simple trilateration algorithm to estimate its own position. A *qualified node* is calculated with at least three position packets, resulting in a more accurate position estimation, as shown in Figure 1a. However, even with only one or two samples, it is still possible to obtain a rough estimate. With only one sample, the sensor node assumes that it is at the same position as the sink. With two samples, it considers its position as the midpoint between the intersection of the position packets’ circles. The proposed ECKN is able to maintain similar network performance (throughput, overhead) when compared to the state-of-the-art benchmark approach while localizing nodes and extending the network lifetime simultaneously. The main difference between the proposed ECKN and the existing works found in the literature is the fact that it is a joint localization, routing, and energy-aware mechanism. Other existing works take each of these issues separately, or at most two, simultaneously, e.g., localization and routing. ECKN demonstrates that it is possible to extend the wireless sensor the network lifetime while performing localization and proper routing.

Although trilateration is a viable solution for the localization problem, there are some issues that need to be addressed. GPS location is not 100% accurate due to the noise that creates some uncertainties in its estimations [9]. When the sensor node receives position packets and calculates the intersection between them, it is not always able to find a common point where they intersect. Instead, it ends up with six different intersection points, as shown in Figure 1b. Using this information, the sensor node has to estimate its position by deciding which intersection points, called *reference points*, are the best to be used in the estimation. Furthermore, in the worst-case scenario where the sink moves randomly within the WSN boundary, some position packets might not be ideal for performing trilateration. Figure 1c shows an example of poor samples, in which collinear position packets are received by a sensor node, making it impossible for the trilateration algorithm to estimate an accurate position.

In a WSN, communication between the source and destination nodes may require a multi-hop communication strategy due to constraints on the radio range and other resources. As mentioned earlier, nodes can properly forward messages to their destinations by being aware of their positions. This information is widely used to improve the performance of routing protocols. A popular approach to this routing issue is geographic routing.

**Figure 2:**
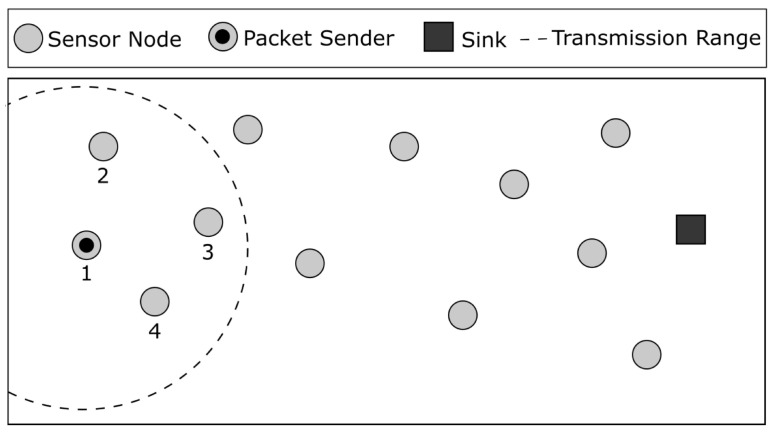
Greedy geographic forwarding example. Node 3 is going to receive the packet because it is closer to the sink.

Geographic routing is one of the most popular routing schemes in WSNs because it scales well, meaning that network size is not a problem with this scheme. The forwarding decision is based on the position of the node and its neighbors. The most straightforward algorithm for geographic routing is greedy geographic forwarding (GGF). The idea behind GGF is to forward a packet to the neighbor node that is closer to the destination in terms of Euclidean distance based on their position coordinates. Figure 2 shows an example of greedy geographic forwarding, where node 1 receives a packet destined for the sink and then forwards that packet to node 3, which is the closest neighbor to the destination. If there is no neighbor that is closer to the destination, GGF fails, and the packet is dropped.

Research efforts have focused on duty-cycled approaches [3,10,11] aimed at improving the network lifetime while maintaining a high delivery guarantee. Guaranteed delivery refers to the success ratio of forwarding a message from the source to the destination, which requires that there is at least one path connecting these two nodes in the network. Existing works on duty cycling aim to achieve network coverage and/or connectivity by finding the best nodes to sleep in order to minimize the number of awake nodes while maintaining network coverage and/or connectivity.

This journal presents energy-aware connected k-neighborhood (ECKN), a holistic solution that integrates position estimation, packet routing, and sleep scheduling in wireless sensor networks. We observed that these processes share some common features and can benefit from using the sensor node positions. Specifically, our approach leverages the sink’s position broadcast for packet routing and enables nodes to estimate their positions based on this information. Moreover, our sleep scheduling algorithm aims to maintain network connectivity to facilitate successful packet delivery. By integrating these functionalities, our goal is to enhance the network lifetime by minimizing any overhead that may arise.

The remainder of this paper is structured as follows: Section 2 provides an overview of related work in this area. Section 3 details our proposed algorithm for position estimation. Section 4 presents the packet routing algorithm. Section 5 describes the sleep scheduling algorithm we propose. The performance criteria and experimental results obtained via simulation are presented in Section 6. Finally, Section 7 concludes the paper.

## 2. Related Works

Over the last decade, the scientific community has proposed numerous research works related to sensor node position estimation, packet routing, and duty cycling in WSNs.

### 2.1. Sensor Node Position Estimation

Existing efforts in sensor node position estimation can be organized into two categories: range-based and range-free. Range-based approaches [12,13] assume that sensor nodes can measure the range or distance between them. Range-free approaches [14,15] use nondeterministic attributes of the network, such as closeness and hop count, to perform position estimation. Range-free approaches usually show lower accuracy than range-based solutions.

An adaptive range-based trilateration localization algorithm (ARBL) is proposed in [12] in order to improve the accuracy of range-based trilateration localization for WSN nodes in outdoor conditions. The algorithm is based on reference node selection and aims to find the best reference nodes at a given time for computing the final location estimate of a node, taking into account the prevailing localization geometry and ranging errors. Panday and Varma [13] proposed a cooperative range-based localization system, where they modified a range-based algorithm in order to save energy by reducing collisions and retransmission of range packets.

R. Mani et al. [14] proposed an approach that uses a bounding box method to identify the possible location area of unknown nodes, and a Kalman filter to refine the localization process and improve accuracy. The focus is on decreasing the number of anchors and reduceing costs. The range-free method is used to estimate inter-distance via the calculation of the lowest number of hops, and the mobile anchor node trajectory could be random or predetermined. Chen et al. [15] proposed ZBLM and EZBLM, two range-free localization methods that utilize only two anchor nodes, placed on the bottom-left and bottom-right corners of a square region of the WSN, and use bilateration to estimate node positions by counting the minimum number of hops to the anchor nodes.

### 2.2. Packet Routing

Greedy perimeter stateless routing (GPSR) is a well-known routing algorithm proposed by Karp et al. (2000) [16]. Nodes determine their neighbors’ positions by receiving periodic beacons. To reduce beaconing costs, GPSR piggybacks the local node’s position in data packets it forwards, and each node receives a copy of all packets for nodes within the radio range. GPSR uses the GGF algorithm to send packets to the destination nodes, and if it fails, a perimeter forwarding algorithm is used to exploit the faces of a planar graph of the network topology. To avoid crossing links, GPSR removes edges that are not part of the relative neighborhood graph (RNG) or Gabriel graph (GG), which are two planar graphs known in the literature [17,18]. Using the planar graph, GPSR traverses the edges of a face using the right-hand rule until it reaches a node closer to the destination.

An improved GPSR algorithm is proposed in [19]. It uses surplus energy to send a signal to a distant node instead of transmitting a message. The method involves using an aggregate node located at the remote base station to route messages through proper nodes. The network operation involves selecting headset users for groups and transmitting data from the users to the base station. The cluster head node needs to be radioactive, and associate nodes remain in sleep mode. The GPSR system outperforms earlier techniques in terms of routing path lengths.

The face traversal technique (FACE-2) proposed by Bose et al. [20] creates a planar graph based on the network topology and uses face routing to find a path between source and destination nodes. Essentially, the algorithm creates a line segment from the source to the destination, finds a face with the source node on its boundary that intersects this line segment, and traverses this face until reaching an edge that intersects the line segment. When reaching this edge, one of the nodes of the edge becomes the new source node and the process repeats until it reaches the destination node.

Mineno et al. [21] proposed a combined protocol, named OLSR-L, which integrates optimized link state routing (OLSR) [22] and OLSR-based localization (ROULA) [23], which perform routing and localization simultaneously. When running localization and routing separately, it was observed that the communication overhead doubled. To address this, the overlapping functionalities of OLSR and ROULA were integrated to reduce the communication overhead.

OLSR, proposed by Clausen et al. [22], is a proactive routing protocol that uses the concept of multi-point relay (MPR) nodes, which forward broadcast messages during the flooding process. To select MPR nodes, the OLSR algorithm floods each node’s 1-hop node list to their 1-hop nodes and selects MPR nodes when the node has the same 2-hop node list. Then, the selected MPR nodes act as relay nodes to form the routing table.

ROULA, proposed by Takenaka et al. [23], is a range-free and anchor-free localization protocol where nodes search for other nodes arranged into regular triangles to satisfy the requirement of independency from anchors. To choose the farthest 2-hop node, which is a candidate to be a vertex of a regular triangle, ROULA uses MPR nodes. Each node then floods the network with packets carrying a farthest 2-hop node list. Based on this information, nodes calculate relative local coordinates by matching regular triangles. Therefore, OLSR-L performs localization and routing simultaneously, with OLSR periodically holding and updating 1-hop neighbor information to provide ROULA’s required neighbor node information. Furthermore, ROULA uses OLSR’s MPR node to accurately estimate node distance.

Oliva et al. [24] proposed a solution for simultaneous routing and localization in multi-hop wireless networks. The solution involves clustering and routing steps where nodes are collected into clusters using the CBRP algorithm [25]. During this step, kernel nodes send their position to neighbors for localization. The shadow edges localization procedure is performed during the cluster localization step for clusters having more than three non-collinear, connected localized nodes. For clusters having less than three non-collinear, connected localized nodes, the super-cluster localization step is used, which considers nodes in adjacent clusters. The shadow edges localization procedure is applied to the gateway nodes to improve the overall position information.

Kirci et al. [26] proposed a system that, when a mobile node needs to calculate its position, it attempts to use reference nodes that are one hop away. If it does not have enough reference nodes that are one hop away to calculate its position, then it looks for multi-hops-away reference nodes that will serve as distance reference nodes. To do so, the sensor node uses its neighbors that can relay using the OLSR [22] by routing the information of other ad hoc nodes that are multi-hops away from the sensor node. When a distance reference node is found, the estimated distance to the sensor node is calculated using the DV-hop and DV-distance methods. The ad hoc routing is necessary to search the multi-hop node that knows its position, and the OLSR routing is used to offer the position calculation of the nodes. Finally, the recursion-based localization process will be completed by offering to estimate distances of nodes that are multi-hops away from the first node that already calculated its position.

Cota-Ruiz et al. [27] presented a routing algorithm that can be used in the field of centralized range-based localization schemes. Given two non-neighboring sensors, an unknown sensor and an anchor node, and only using the network connectivity, all of the evaluated shortest paths with the minimum number of hops are averaged to obtain a final distance estimate. This process can be repeated with different anchor nodes in order to estimate the unknown node position using both distance estimates and the absolute positions of anchors. To estimate distances between two non-neighboring sensors, the proposed algorithms follow two steps: to find all paths with minimum hops between the non-neighboring sensors and to obtain the length of the paths based on the known one-hop distance. The non-neighboring distance estimate is calculated as the mean of all evaluated path distances.

### 2.3. Duty Cycling

Duty cycling in WSN can be achieved in two different and complementary approaches: topology control and power management. Topology control approaches [28,29,30] exploit node redundancy, selecting a minimum subset of sensor nodes to remain active for maintaining connectivity. On the other hand, power management approaches [31,32] refer to active nodes switching off the radio when there is no network activity, alternating between sleep and awake periods.

Tolani et al. presented in [10] an energy-efficient medium access control protocol that optimizes the turn-on and -off times of transceiver radios based on slot utilization estimation. The proposed duty cycle approach is shown to consume less energy than existing protocols through mathematical and simulation analyses. Both cluster head and sensing devices save energy by periodically checking the buffer status and turning off radios when the buffer is empty.

Ren et al. proposed an opportunistic routing algorithm with dynamic transmission power and duty cycling for energy harvesting in WSNs [11]. It adjusts the transmission power of each node based on predicted available energy and energy utilization in the previous slots. The algorithm also updates relay sets and forwarding paths to reduce delays and retransmissions. The proposed scheme aims to decrease retransmission and delay caused by heterogeneous transmission power/radii of nodes. The study introduces an improved transmission model and a novel information exchange mechanism for dynamic updates.

Connect k-neighborhood (CKN) is an algorithm proposed by Nath and Gibbons [33] to keep at least min(k,d) nodes awake in a sleep scheduling approach, where *k* is the number of connected nodes and *d* is the degree of each node. A sleep scheduler selects a subset of nodes to remain awake in a given epoch, the remaining nodes are set to a sleep state. The subset of awake nodes changes from epoch to epoch in order to improve the network lifetime. The proposed algorithm generates a connected graph every epoch, enabling nodes to send and receive packets from each other within the network.

Span is a topology control protocol proposed by Chen et al. [28], nodes running Span make local decisions on whether to sleep or stay awake as a coordinator, performing multi-hop routing. To determine if a non-coordinator node should become a coordinator or not, the node uses the following coordinator eligibility rule: using information gathered from local broadcast messages, a non-coordinator node checks if two of its neighbors cannot reach each other either directly or via one or two coordinators; if that is the case, the node should become a coordinator in order to maintain network connectivity. The coordinator withdraw phase follows the same idea, a node checks if it should withdraw as a coordinator if every pair of its neighbors can reach each other either directly or via one or two other coordinators. According to the authors, Span preserves network connectivity and capacity, it also decreases latency, while providing significant energy savings.

Cerpa et al. [29] proposed adaptive self-configuring sensor networks topologies (ASCENT). A node running ASCENT decides whether to become active or continue to sleep based on information about connectivity and packet loss that are measured locally by the node itself. Initially, there are only a few active nodes in the network, the remaining nodes keep listening but not transmitting messages. When the sink experiences significant packet loss from the source, it starts sending help messages to signal passive neighbors to join the network. Upon receiving a help message, the passive node decides if it is going to join the network. As soon as it decides to join, the node signals the existence of a new active neighbor to the other passive nodes and it starts transmitting and receiving packets. This process repeats until the packet loss is reduced. ASCENT limits the packet loss due to collisions because the node density is taken into account as a parameter and has good scalability properties.

Sparse topology and energy management (STEM) is a technique proposed by Schurgers et al. [31]. It was noticed that (most of the time) the network only monitors the environment in anticipation of an event occurring. Therefore, STEM uses two different radio signals. Each node periodically turns on its wakeup radio for a short time to listen if there is any neighbor attempting to communicate with it. When a source node needs to communicate with a neighboring node, it sends a stream of beacons on the wakeup channel. Upon receiving a beacon, the target node sends a wakeup acknowledgment and turns on its data radio. The actual data packets are transmitted through this radio. After the data transmissions have ended, the node turns its data radio off again. Results show that STEM can reduce the energy consumption of the network; however, it also increases the setup latency.

A pipelined tone wakeup (PTW) scheme for WSN is proposed by Yang et al. [32]. Similar to STEM, PTW works with two different channels for transmitting wakeup beacons and the actual data packets. The difference relies on what happens after receiving wakeup beacons and the wakeup procedure is pipelined with the packet transmission. When a node receives a beacon, it sends a wakeup acknowledgment and turns on its data radio; at the same time, the receiver node will send a wakeup beacon to wake up all its neighbors. The pipeline process reduces the wakeup latency and, consequently, the overall message latency.

The main difference between the proposed ECKN and the existing works found in the literature is the fact that it is a joint localization, routing, and energy-aware mechanism. Other existing works take each of these issues separately, or at most two, simultaneously, e.g., localization and routing.

## 3. Sensor Node Position Estimation

In a WSN, where a mobile sink collects information from sensor nodes, it is common for the sink to be out of range for nodes that have packets to forward. As a result, the forwarding node needs to find a path through other sensor nodes to reach the sink. Location awareness can help the node decide on the best neighbor to forward the packet to. By knowing the positions of its neighbors and the sink, the forwarding node can send the packet to a neighbor that is closer to the sink. However, using a GPS module is not feasible for small, cheap, low-power devices similar to ordinary wireless sensor nodes. To address this, the sink periodically shares beacons with its new position as it moves, allowing sensor nodes to have up-to-date position information for position-based packet routing. Sensor nodes can also use these position packets to estimate their own positions.

**Figure 3:**
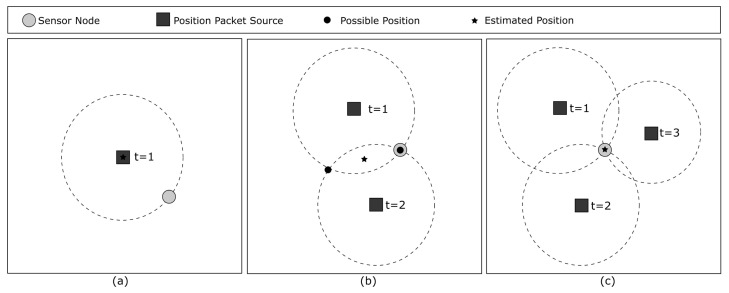
Overview of how the position of the sensor node is estimated. (**a**) With one position packet source. (**b**) With two position packet sources. (**c**) With three position packet sources.

In the proposed position estimation algorithm, a sensor node can estimate its own position using packets from both the sink and neighboring nodes. Once a node estimates its position, it shares it with neighbors to help them estimate more accurate positions. It is important to note that position information from the sink is more reliable because it is based on the GPS position. Therefore, a sensor node will always prefer position information from the sink over information from a neighbor node. Although at least three position data points are needed for more accurate estimation, it is still possible to estimate a node’s position using only one or two position data points, as shown in Figure 3.

The estimation of a node’s position can be performed according to the number of position data points (or samples) it receives from position sources:

- One position sample: The sensor node will assume that its position is the same as the one received in the packet. It is not accurate, but with this information, it is possible to at least determine the region where an event occurred.
- Two position samples: When calculating the intersection between two position packets, we have two possible positions for the node. Since we do not have any other information, the approximate position of the sensor node is the midpoint between these two calculated positions.
- Three position samples: Now a node can estimate a more accurate position and it becomes a *qualified node*. Using the trilateration algorithm, it is possible to estimate the sensor node position by finding the intersection between these three position samples.

At the start of network operation, the positions of sensor nodes will be inaccurate due to the lack of position packets required for estimating node locations. However, over time, node localization will become increasingly precise as more position packets are disseminated in the sensor field as the sink moves. Subsequently, the network will be able to track events occurring in the area and forward them to the sink.

### 3.1. Uncertainties in GPS Positioning

**Figure 4:**
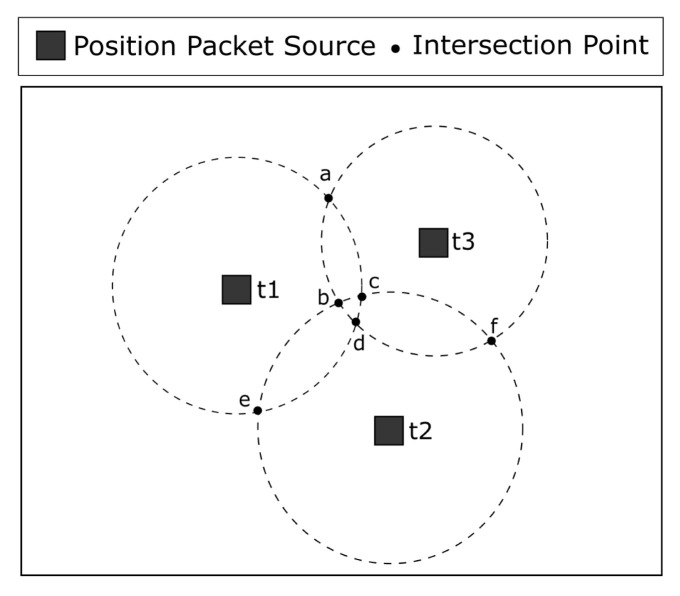
Intersection Points among the Position Packets.

Although trilateration is a viable solution for the localization problem, GPS uncertainty makes position estimation more complex. When receiving position packets and calculating the intersection between them, there is no common intersection point among the packets due to the GPS error. Instead, each intersection will generate two intersection points and the sensor node will have to decide which one to use as a reference point to estimate its position. Figure 4 shows an example of how a sensor node calculates the intersection points. It receives three position packets from the sink at times t1, t2, and t3. The intersection between t1 and t2 generates the intersection points *c* and *e*. The intersection between t1 and t3 generates the intersection points *a* and *d*. Similarly, the intersection between t2 and t3 generates the intersection points *b* and *f*. We propose two algorithms to find out which intersection points to use as reference points: distance and closeness algorithms. Afterward, the sensor node can simply estimate its position, calculating the average of the reference points (a,b,c) obtained according to Equation (1).

(1) $$P\left(x,y\right)=\left(\left(x_{a}+x_{b}+x_{c}\right)/3,\left(y_{a}+y_{b}+y_{c}\right)/3\right)$$

#### 3.1.1. Distance Algorithm

In order to obtain the reference points through the distance algorithm, the sensor node calculates the distance between each pair of intersection points. For instance, consider the set of pairs (c,e), (a,d), and (b,f) of Figure 4. When deciding between points *c* and *e*, the sensor node calculates their distances to all other intersection points, which are *a*, *b*, *d*, and *f*. The point with the smallest distance (point *c* in the example) is set as a reference point, as can be seen in Equation (2).

(2) $$\begin{array}{c} \forall p\in s:min(\sum (d(i,a)+d(i,b)))\;\forall o(a,b)\in s\;and\;o\neq p \end{array}$$

where *p* represents the pair of intersection points, *s* denotes the set of pairs, d(x,y) represents the distance between intersection points *x* and *y*, *i* represents each intersection point of the pair, *o* denotes the other pair of intersection points *a* and *b*.

#### 3.1.2. Closeness Algorithm

Our second algorithm used to obtain the reference points is called closeness. In this algorithm, the sensor node creates every possible combination among the intersection point pairs. In Figure 4, the sets created after this combination are (a,b,c), (a,b,e), (a,c,f), (a,e,f), (b,c,d), (b,d,e), (c,d,f), and (d,e,f). Subsequently, the sensor node calculates the distance between all pairs of points in each set. Then, it sums the distances within a set. The intersection points in the set with the smallest sum are considered as reference points, as can be seen in Equation (3).

(3) $$\begin{array}{c} rp=ip(a,b,c):ip\in s\;and\;ip\;has\;min(d(a,b)+d(a,c)+d(b,c)) \end{array}$$

where rp represents the set of reference points, ip denotes each combination of three intersection points (*a*, *b*, and *c*), *s* represents the set of these combinations, and d(x,y) represents the distance between two intersection points *x* and *y*.

### 3.2. The Position Packet Validation Algorithm

**Figure 5:**
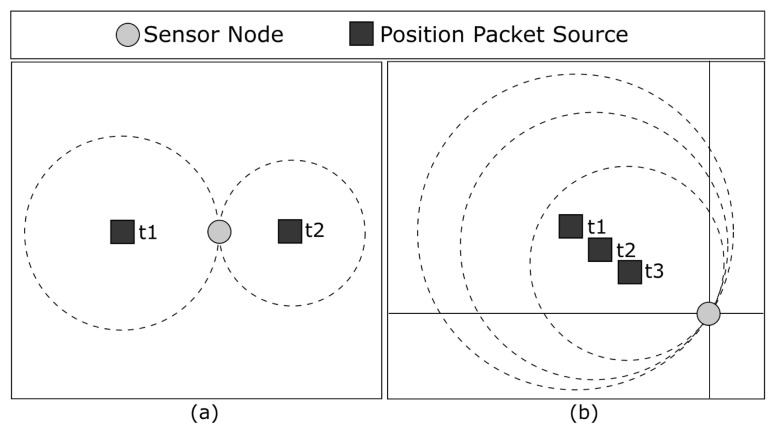
(**a**) Position packets in opposite sides may not intersect due to the GPS error. (**b**) Poor trilateration due to all position packets laying on the same quadrant.

Since the mobile sink is more powerful, it can carry a GPS module. Thus, it can provide position packets to sensor nodes in order to aid them in estimating their own positions. Furthermore, in our scenario, the random mobility adopted by the sink implies that some position packets might not be good enough to be used in the trilateration algorithm, as can be seen in Figure 5. Figure 5a shows a sensor node receiving two position packets at times t1 and t2 from opposite directions. In this scenario, the intersection between these packets generates a single point. However, a GPS error may occur and it may shift t1 slightly to the left and/or t2 slightly to the right. In this case, the circles would not intersect, making it impossible to estimate the sensor node position. Thus, upon receiving a new position packet, the sensor node will first validate if this packet position circle intersects with the positions previously validated. If so, the next phase of the validation will be performed. Otherwise, the packet is discarded.

In Figure 5b, it can be observed that all position packets shown belong to the same quadrant when the network area is divided into four quadrants with the sensor node positioned at the center. This results in poor position estimation due to the GPS error, which produces a small deviation that leads to a large discrepancy of intersection points when one circumference is contained within another. Therefore, we propose three algorithms to validate the position packet and avoid the scenario described above: position distance, circle limit, and a hybrid solution. Once the position packet is validated and accepted, i.e., the position is a good candidate to be used in the trilateration algorithm, the sensor node stores its information for future estimations.

Even after receiving three good candidate position packets, the sensor node continues to receive such packets in an attempt to improve its position estimation. Specifically, it uses the received signal strength of a newly received packet to calculate the distance between its estimated position and the source of the packet. If these values do not match (within a certain threshold), the sensor node validates the packet and checks whether replacing one of its current position packets with the new one will result in a more accurate estimation. If this is the case, the replacement is made permanently.

#### 3.2.1. Position Distance Algorithm

When executing the position distance algorithm, a sensor node stores the information of the first received position packet. As soon as the next position packets are received, the sensor node calculates the distance between the source position of the newly received packet and the source position of each packet that was already stored. If all of these distances exceed a predefined threshold (i.e., Equation (4) is true), the packet is accepted and the sensor node stores its information to be used in future estimations. If at least one of the calculated distances does not exceed the threshold, the received position packet is considered invalid and it is dropped.

**Figure 6:**
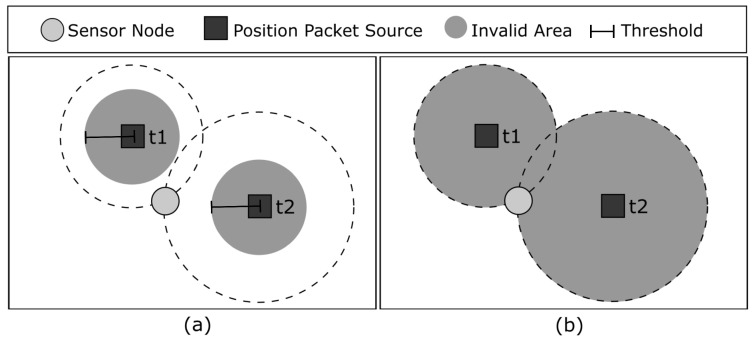
(**a**) Invalid area for position packets according to the position distance algorithm. (**b**) Invalid area for position packets according to the circle limit algorithm.

Figure 6a shows a sensor node that received, validated, and stored two position packets at times t1 and t2. The shaded region represents the area where other position packets will be considered invalid based on the threshold. This is done to avoid the issue depicted in Figure 5b, where all the received position packets belong to the same quadrant, leading to poor position estimation due to GPS error. Any packet received from a position outside this area will be accepted.

(4) ∀p∈rp:d(p,r)>t

where rp represents the set of received packets, *r* is the newly received packet, d(p,r) is the distance between *p* and *r*, and *t* is the predefined threshold.

#### 3.2.2. Circle Limit Algorithm

When using the hybrid algorithm, a sensor node combines the validation rules of the position distance and circle limit algorithms. Upon receiving a new position packet, the sensor node checks whether the source position of the received packet is within the circle of each stored packet, and calculates the distance between the source position of the received packet and the source position of each stored packet. If either of these conditions is satisfied, the packet is considered invalid and dropped. Otherwise, if all of the calculated distances exceed the predefined threshold and the source position of the received packet is not within any of the circles, the packet is accepted and the sensor node stores its information to be used in future estimations. Figure 6c shows a sensor node that received, validated, and stored two position packets at times t1 and t2. The shaded regions represent the circles calculated based on the received signal strength, and the dashed line represents the threshold for the distance validation. If the received position packet is within the shaded area or its circle contains one of the validated source position packets, or the distance between the received packet and the stored packets is less than the threshold, it is considered invalid. Otherwise, the received packet is accepted.

(5) $$\forall p\in rp:r\notin c_{p}\;\mathrm{and}\;p\notin c_{r}$$

where rp represents the set of received packets, *r* is the newly received packet, $c_{p}$ and $c_{r}$ are the circles of *p* and *r*, respectively.

#### 3.2.3. Hybrid Algorithm

The position distance algorithm improves the probability of a quadrant containing a single position packet but does not guarantee it. The circle limit algorithm, on the other hand, guarantees that a quadrant will have only one position packet, but reduces the chances of validating and accepting new position packets. This means that a sensor node may take longer to estimate an accurate position. Therefore, we propose a hybrid algorithm that combines both position distance and circle limit algorithms. Initially, a sensor node will use position distance until it has received and validated three position packets. Once the node is considered qualified, it switches to the circle limit algorithm to improve its position estimation. In this way, a node can quickly estimate an acceptable position initially and then improve it over time.

## 4. Event Packet Forwarding Algorithm

**Figure 7:**
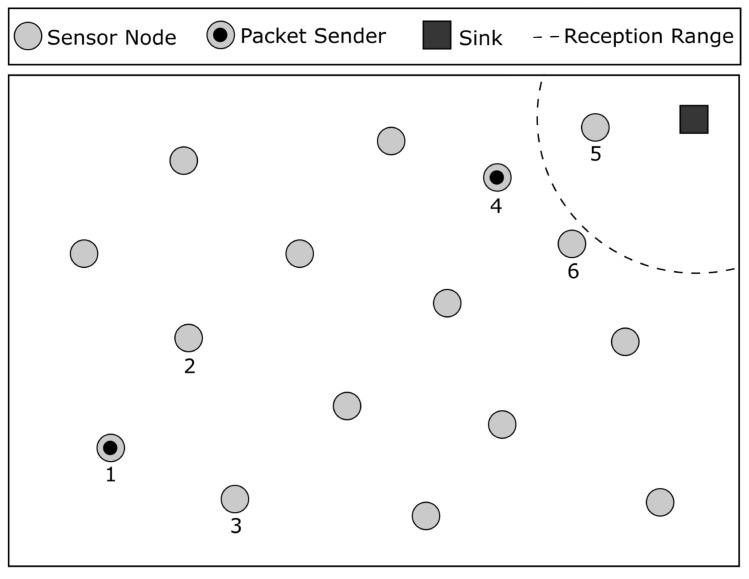
Distance to sink. Sensor node 1 can choose between 2 and 3 and the distance is not a great factor to make the decision. On another hand, it is more important that node 4 forwards the packet to sensor node 5 than to sensor node 6 because node 5 is in the sink reception range.

The proposed packet forwarding algorithm aims to determine the optimal node to forward a packet based on the GGF [16] algorithm. This is achieved by using a utility function that takes into account various network characteristics, such as the power level and distance to the sink. Power level refers to the amount of energy a sensor node has remaining. Nodes with more energy are better suited to forwarding the packet as they are less likely to shut down before transmitting to the next hop. The distance to the sink represents the ratio between the distance from the receiving node to the sink and the distance from the sending node to the sink. The lower this ratio, the better the node is for packet forwarding as it will be closer to the sink. This ratio is particularly important when the packet is near the sink, as there may be multiple routes available for forwarding the packet. An example of this scenario can be seen in Figure 7.

After the localization estimation algorithm runs, each sensor node stores the position of its neighbors and the sink. As the sink moves, it shares its new positions, and the sensor nodes update the stored sink position to ensure they have the most recent information. The sink position packet also has a time to live (TTL), which means that when a sensor node receives this packet, it decreases the TTL by one. If the TTL is still greater than zero, the node forwards the packet to its neighbors, allowing nodes further away from the sink to update their sink position information. Table 1 provides a description of the fields found in the sink position packet. Similarly, each sensor node shares its current battery level, its address, its position, and the accuracy of its position, which is determined by using three accurate position packets. Table 2 provides a description of the contents of a sensor node position packet.

Using this information, it is possible to define a utility function expressed by Equation (6) to estimate the best node to forward the packet to.

(6) $$U_{i}=w_{p}P_{i}+w_{d}\left(1-D_{i}/D\right)$$

(7) $$w_{p}+w_{d}=1$$

where *i* is the *i*th neighbor node, $w_{p}$ is the weight for the power attribute, $P_{i}$ is the power of neighbor nodes *i*, $w_{d}$ is the weight of the distance attribute, $D_{i}$ is the distance to the sink of the neighbor node *i*, *D* is the distance to the sink of the sender node.

By Equation (7), we have that the sum of the weights must be 1. Since the routing algorithm is based on GGF, the most important attribute in Equation (6) is the distance to the sink. Thus, the proposed values for the weights are $w_{p}$ = 0.25 and $w_{d}$ = 0.75, in order to prioritize the sensor node position.

### Sink Position Bridges

The proposed packet forwarding algorithm relies on the sink position, which means that sensor nodes must be aware of the current position of the sink and update it as the sink moves.

As described in Section 4, the sink broadcasts its position along with a time-to-live (TTL) value. When a sensor node receives this packet, it decreases the TTL by one and checks if it is still greater than zero. If the TTL is positive, the node forwards the packet to its neighboring nodes.

**Figure 8:**
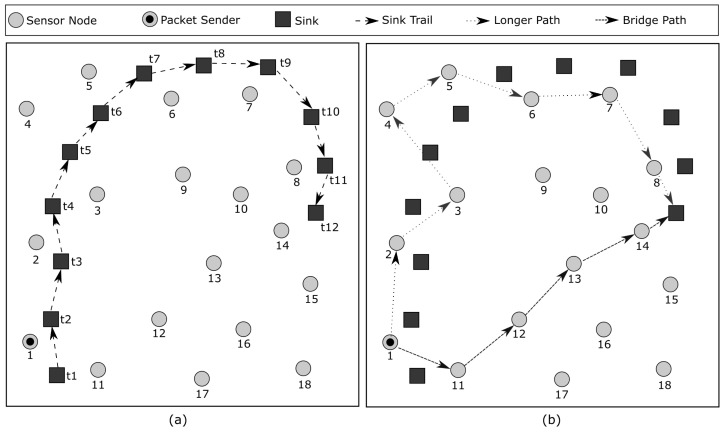
(**a**) Bridges depend on the sink movement. (**b**) A bridge is established when the sink determines that there exists a shortest path between its current position and a previous position exists.

Since the sink has random mobility, it is possible that an event packet travels a much longer path to reach the sink depending on how the sink moves. Figure 8b shows one example of this path. Sensor node 1 will forward the packet following the sink trail; thus, the path that the event packet will take to reach the sink is 1→2→3→4→5→6→7→8. However, there is a possible shortest path for this event packet, which is 1→11→12→13→14.

In order for the sink to find the shortest path, i.e., bridge, between its previous positions and its current position, the sink keeps track of a certain number of previous positions to estimate the existence of a bridge. As the sink moves, previous positions are stored so that its traveled distance can be estimated. For instance, Figure 8a shows where the sink saves its position, which is conducted every *x* meters. When the sink is at t12, it can easily calculate how much it has traveled. For instance, when the sink is at t11, it has traveled *x*; at t10, it has traveled 2x; at t9, is has traveled 3x, and so on.

Once the distance that the sink has traveled is determined, we can compare the Euclidean distance between its current and previous positions. If the ratio of these two distances is less than a predetermined threshold, it indicates that there is a shortest path, and a bridge needs to be created.

To create a bridge, the sink sends its position packet with a larger TTL value, which means that this packet will reach the sensor nodes that are close to its previous position. This helps those nodes to update the sink position and find the shortest path.

## 5. Sleep Scheduling Algorithm

**Figure 9:**
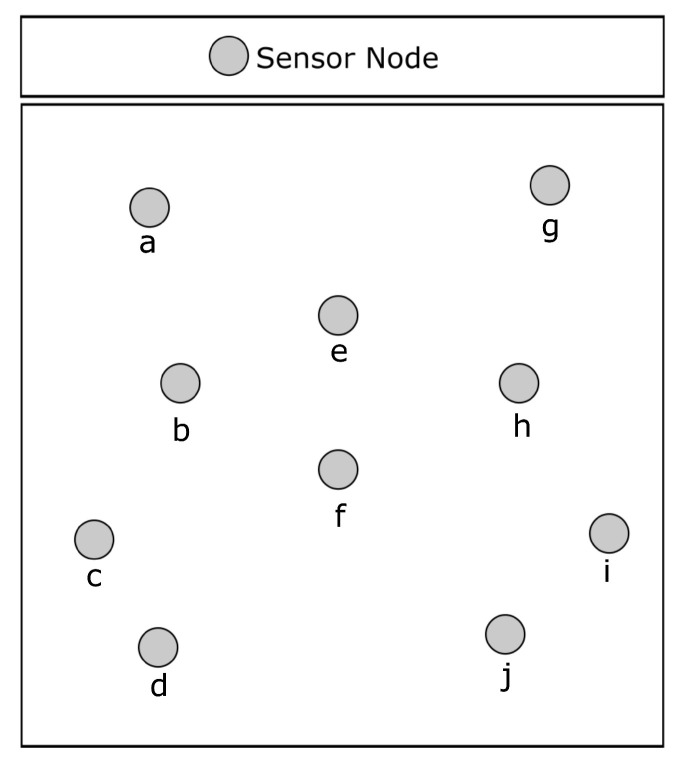
Sleep scheduling may disconnect the network if nodes *e* and *f* go to sleep in the same epoch.

Network lifetime is a critical aspect of wireless sensor networks (WSNs). Due to the limited power of sensor nodes, several techniques have been explored to prolong network longevity, one of which is sleep scheduling. This method involves sensor nodes working in duty cycles, with different nodes sleeping in each epoch while the rest remain awake. During sleep periods, nodes reduce their transmission and reception channels and processing power to conserve energy. However, sleep scheduling may impact packet routing and cause network disconnection depending on which nodes go to sleep. For example, in Figure 9, nodes *e* and *f* act as the only connection between nodes *b* and *h*. If both nodes *e* and *f* go to sleep simultaneously, there will be no route between nodes *b* and *h*.

In order to avoid scenarios such as the one described above, Nath et al. [33] proposed an efficient decentralized sleep scheduling algorithm to reduce the number of awake nodes while maintaining the network connected. Their algorithm addresses the connected k-neighborhood (CKN) problem, an NP-complete problem in graph theory: Given a constant *k* and an undirected graph G=(V,E), find a subset of nodes C⊂V, such that *C* is a minimum connected k-neighborhood. In CKN, (i) each node v∈V has at least $m=min(k,d_{v})$ neighbors from *C*, where $d_{v}$ is the degree of *v* in the *G*, and (ii) the nodes in *C* are connected. *C* is a minimum CKN if no CKN has a smaller number of nodes.

The near-optimal solution to the CKN problem is presented in Algorithm 1, with a detailed explanation provided in the work by Nath et al. [33]. To determine whether a node will go to sleep, each node selects a random rank from a random number generator, which is then compared with its neighborhood. Nodes with larger ranks have a higher probability of going to sleep. However, to balance the network lifetime among nodes, Yuan et al. [34] proposed using the residual energy information of the nodes to determine whether a node will be awake or asleep. In our solution, we take advantage of the energy information shared for packet routing to obtain the rank for each node using Equation (8). Once the rank is determined, the sensor nodes use the same algorithm proposed by Nath et al. [33] to estimate whether they will stay awake or go to sleep, skipping step 1 of the algorithm since the rank is already known.

(8) $$rank_{u}=1-energy_{u}$$

where $rank_{u}$ is the rank that node *u* will use to determine if it is going to remain awake or go to sleep, and $energy_{u}$ is the residual energy of node *u*.
**Algorithm 1** Connected k-neighborhood (CKN) (* run the following at each node u *).1:Pick a random rank $rank_{u}$.2:Broadcast $rank_{u}$ and receive the ranks of its currently awake neighbors $N_{u}$. Let $R_{u}$ be the set of these ranks.3:Broadcast $R_{u}$ and receive $R_{v}$, from each $v\in N_{u}$.4:If $|N_{u}|<k$ or $|N_{v}|<k$ for any $v\in N_{u}$, remain awake. Return.5:Compute $C_{u}=\left\{v|v\in N_{u}\mathrm{and}rank_{v}<rank_{u}\right\}$6:Go to sleep if both of the following conditions hold. Remain awake otherwise.Any two nodes in $C_{u}$ are connected either directly or indirectly through nodes within *u*’s 2-hop neighborhood that have a rank lower than $rank_{u}$.Any node in $N_{u}$ has at least k neighbors from $C_{u}$.
7:Return.

It has been observed that sensor nodes near the sink consume more energy since they receive packets from the entire network for forwarding them to the sink. In contrast, nodes far from the sink consume less energy due to the lower packet traffic. Based on this observation, we propose different values for parameter *k* depending on the distance of a sensor node from the sink. We divide the network into three regions: high, medium, and low traffic. Nodes in the high-traffic region will have a higher value of *k* since they receive packets from all three regions (high, medium, and low), which means that more nodes will stay awake in this region and the workload can be distributed. Nodes in the medium-traffic region will have a medium value of *k* since they receive packets from medium- and low-traffic regions only. Nodes in the low-traffic region will have a smaller value of *k* since they are far from the sink and receive packets only from nodes in the low-traffic region. In this region, more nodes can go to sleep to conserve energy.

**Figure 10:**
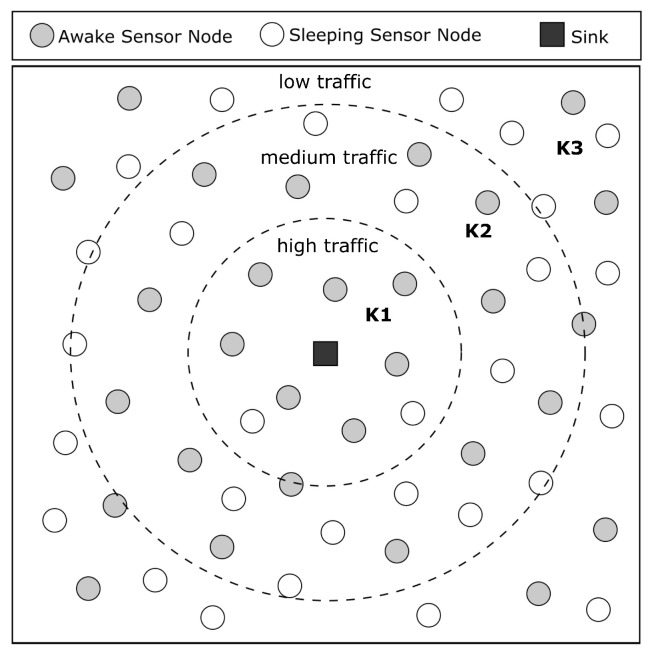
The closer the nodes are to the sink, the higher the value of K. In this way, the network will be more dense when close to the sink, where there are more packet transmissions.

The previous section presented how the sink and sensor nodes estimate and share their positions. With this information, sensor nodes can determine their region based on the distance to the sink. Figure 10 provides an example of how the network is divided into three regions based on the calculated distances. Nodes in the high-traffic region, which are closer to the sink, have the value k1 for *k*, while nodes in the medium-traffic region have the value k2, and nodes in the low-traffic region have the value k3. The value of *k* increases as the node approaches the sink, i.e., k1>k2>k3. The figure illustrates that the network is denser in the region containing the sink due to the high traffic of packets, while the region further from the sink is less dense due to the lower traffic of packets.

## 6. Performance Evaluation

The proposed schemes were evaluated through an extensive set of simulations to measure their performance. The evaluation was based on several metrics, including position estimation accuracy, packet delivery ratio, and network overhead.

Simulations were implemented in OMNeT++ (OMNeT++ (https://omnetpp.org/ accessed on 1 January 2023) is an extensible, modular, component-based C++ simulation library and framework, primarily for building network simulators) and parameters can be found in Table 3. The network size is 1000 × 1000 m${}^{2}$. The number of sensor nodes varies from 50 to 150 in order to evaluate the schemes in sparse and dense networks. The mobile sink starts at the center of the area, i.e., (500, 500), and its average speed is 10 m/s, e.g., a drone, following the random waypoint as its mobility model [35]. The sink sends its position to nodes every 5 s. The transmission range of the sink and nodes is 185 m using breakpoint path loss [36]. The threshold for the position distance algorithm is 50 m, which presented the best performance. Simulations were run 10 times following the Student’s t-distribution [37]. We compare our algorithms with trilateration without packet validation in order to show performance improvement.

### 6.1. Sensor Node Position Estimation Accuracy

**Figure 11:**
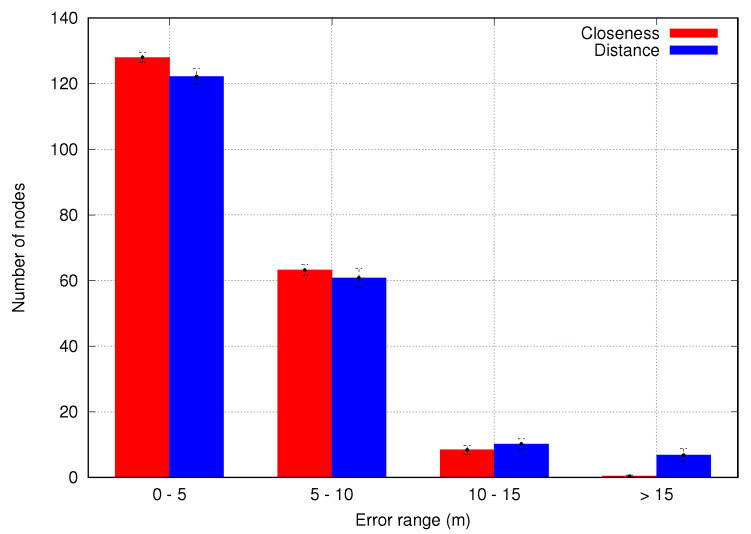
Impact of reference point algorithms in sensor node accuracy.

Firstly, we evaluate the distance and closeness algorithms when selecting the most appropriate reference points. We simulate the same scenario for both algorithms together with the position distance algorithm to estimate node positions. Figure 11 shows the number of nodes within different position estimation error ranges. As can be seen, closeness has more nodes in the small error range while distance exceeds closeness in larger error ranges. Thus, the closeness algorithm outperforms the distance algorithm because it considers all possible combinations of intersection points and chooses the closest points within a combination set to be used as reference points. Therefore, the closeness algorithm is used in the next simulations in order to obtain reference points.

**Figure 12:**
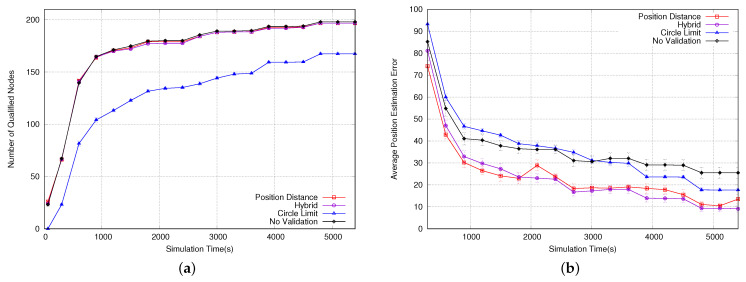
(**a**) Number of qualified nodes. (**b**) Avg position estimation error.

The next simulation evaluated the time it takes for a sensor node to become qualified, as shown in Figure 12a. Both the position distance and hybrid algorithms showed similar performances to that with no validation, and they performed better than the circle limit algorithm. This can be explained in Figure 6, where the position distance algorithm has a smaller invalid area. This means that the probability of accepting a position packet is higher and, consequently, nodes become qualified faster. Additionally, the hybrid algorithm’s performance is similar to that of the position distance algorithm because it uses the same algorithm until the node is considered qualified.

Figure 12b shows the average position estimation error, in terms of the distance between the estimated and actual position for all nodes. The position distance algorithm outperforms the circle limit algorithm because it validates and accepts more position packets. The hybrid algorithm starts to perform better than position distance over time because, after the sensor node is qualified, it starts running the circle limit algorithm, which results in better estimations. Although the algorithm with no validation accepts every position packet, it is outperformed by the other algorithms because it does not avoid situations depicted in Figure 5, resulting in inaccurate estimations.

**Figure 13:**
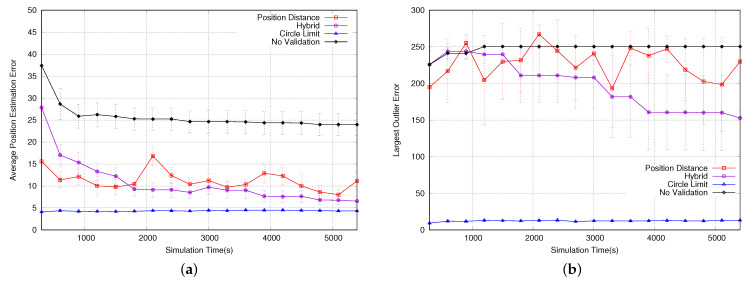
(**a**) Average position estimation error for qualified nodes. (**b**) Largest outlier error.

Although position distance shows better performance in terms of position estimation error when considering all nodes, the circle limit algorithm shows improved performance when we only consider qualified nodes, as shown in Figure 13a. This is because scenarios such as the one depicted in Figure 5b are impossible to occur in the circle limit algorithm, whereas position distance minimizes their occurrences. Hybrid outperforms position distance over time for the same reason that it starts running the circle limit algorithm after having qualified nodes, as discussed earlier. The algorithm with no validation is outperformed once again because it does not avoid situations depicted in Figure 5, which results in inaccurate estimations.

Figure 13b shows the position estimation outlier errors for qualified nodes. The circle limit algorithm demonstrates superior performance by ensuring that each quadrant has only one position packet, leading to better estimations for qualified nodes. The hybrid algorithm shows improved results over time because it eventually switches to the circle limit algorithm. However, the algorithm with no validation and position distance exhibit similar performance, as they both do not completely avoid scenarios, such as the one shown in Figure 5b.

### 6.2. Packet Routing

**Figure 14:**
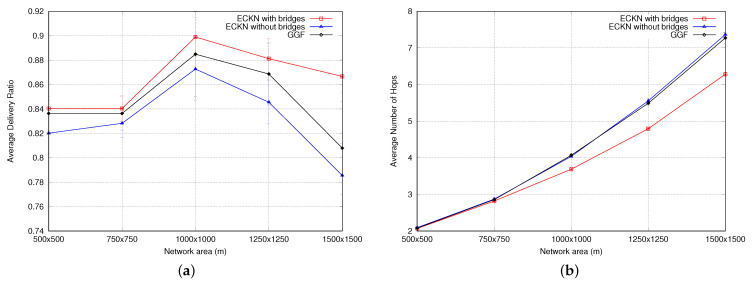
(**a**) Avg delivery ratio, and (**b**) number of hops (for different node densities).

The packet delivery success ratio was evaluated by simulating the protocols in different network densities using 5 different sizes for the network: 500 × 500 m${}^{2}$, 750 × 750 m${}^{2}$, 1000 × 1000 m${}^{2}$, 1250 × 1250 m${}^{2}$, and 1500 × 1500 m${}^{2}$. As shown in Figure 14a, ECKN with bridges outperforms ECKN without bridges and GGF. This is because ECKN creates bridges that shorten the distance between the source node and the sink, increasing the probability of delivering a packet. GGF performs better than ECKN without bridges because ECKN considers not only the positions of the nodes but also their remaining energy, which may cause the packet to take a longer path and increase the probability of dropping the packet. It is also important to note that the 1000 × 1000 m${}^{2}$ network size presents the best average delivery ratio. This is because smaller areas present a high likelihood of collisions, which can result in packet loss during routing. On the other hand, larger areas present blind spots, making routing infeasible and causing event packets to be dropped.

The success of packet delivery is directly related to the number of hops that an event packet takes when traveling from the source node to the sink. Figure 14b shows the comparison between ECKN and GGF. ECKN with bridges presents the smallest average number of hops, which can be explained by the creation of bridges that shorten the path between the source node and the sink, reducing the number of hops needed for the event packet to be delivered. GGF and ECKN without bridges show similar performance, with a slight advantage for the former. This advantage can be attributed to the fact that in GGF, the sensor node always chooses the closest neighbor to the sink to forward the packet, while in ECKN, the remaining energy is also taken into account, which sometimes results in a longer path to reach the sink for ECKN without bridges.

**Figure 15:**
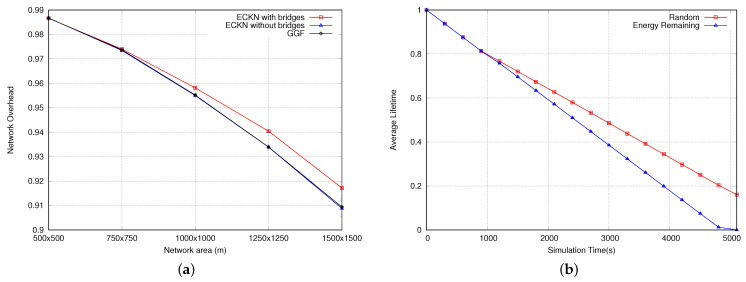
(**a**) Network overhead for different node densities. (**b**) Network lifetime.

The network overhead was also evaluated in our simulations. As shown in Figure 15a, GGF and ECKN without bridges exhibit the same behavior, while ECKN with bridges has a larger overhead. This can be explained by the larger time-to-live (TTL) that ECKN with bridges uses when creating a bridge. While GGF and ECKN without bridges have the same TTL for every position packet from the sink, ECKN with bridges calculates whether it is possible to shorten the path between an area and the position of the sink. If it is positive, it increases the TTL of the position packet to reach a further region. This causes the position packet to be resent more times, increasing the network overhead.

### 6.3. Network Lifetime

In this section, we evaluate the impact of our proposed sleep scheduler on the network lifetime. Firstly, we assess the impact of using the residual energy of the node instead of a random number as a rank in the CKN algorithm. We also evaluate our ECKN algorithm, which has different values of *k* depending on the node’s distance from the sink: k=3 for nodes close to the sink, k=2 for nodes in an intermediate region, and k=1 for nodes further from the sink. We compare our algorithm with the solution that has no sleep scheduler and with CKN having k=3, k=2, and k=1.

Figure 15b shows the network lifetime using random numbers and residual energy as rank. As can be seen, using random numbers as ranks shows better performance than using residual energy. This is explained by how the CKN algorithm works. In order to go to sleep, a sensor node has to follow two conditions depicted in step 6 of Algorithm 1. When we use the residual energy, the node with the largest number in a region will never go to sleep due to the first condition. Since the node has the largest rank, all neighbors will be considered. If two neighbors are on extremely opposite sides, there will not be a two-hop connection between them, and this sensor node will not go to sleep. As the node does not sleep, its rank will increase, and the same problem will occur until the node dies. The same problem happens in different regions of the network causing the lifetime (when using residual energy as rank) to be worse than when using random numbers.

**Figure 16:**
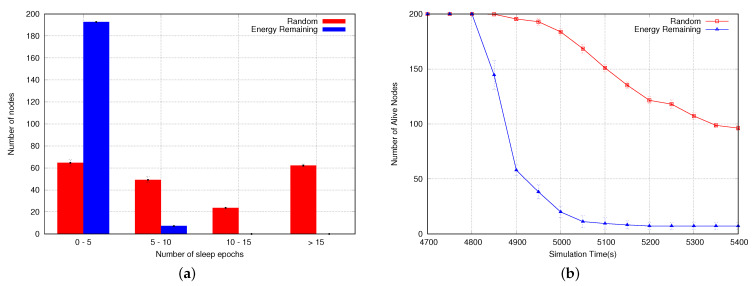
(**a**) Number of sleep epochs using random numbers and residual energy as rank. (**b**) Number of alive nodes using random numbers and residual energy as rank.

Figure 16a shows the number of sleep epochs in which the sensor nodes go to sleep. Results only emphasize what we have mentioned earlier, most of the nodes are sleeping only in 0 to 5 epochs when using the energy remaining as rank. When using random numbers as rank, we have more than 60 nodes having more than 15 epochs where they are sleeping, explaining the better lifetime when using this approach as rank. Figure 16b shows the number of alive nodes at the end of the simulations. As expected, the approach using random numbers as rank for the CKN algorithms presents more alive nodes than the approach with residual energy. These results are the consequence of the problem depicted above regarding the residual energy.

**Figure 17:**
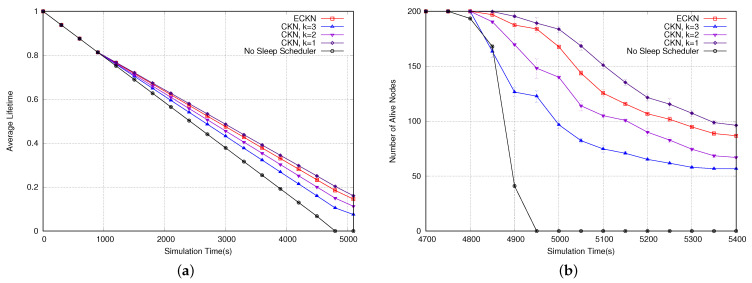
(**a**) Network lifetime and (**b**) number of alive nodes using different sleep schedulers.

**Figure 18:**
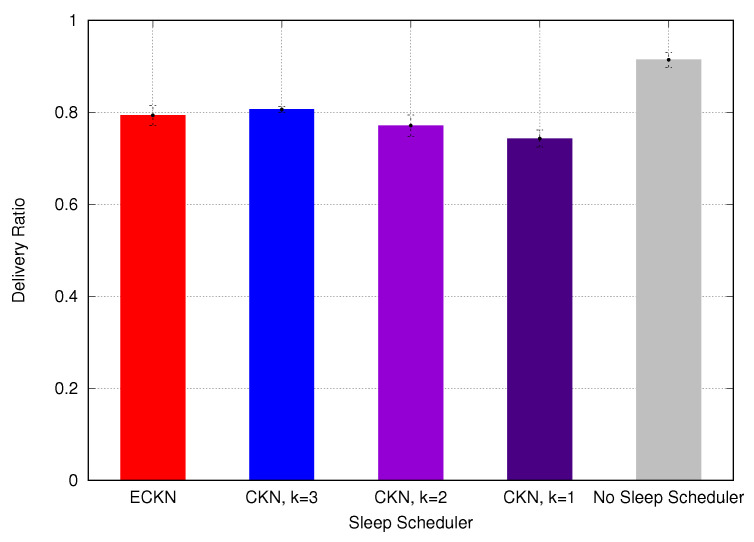
Delivery ratio using different sleep schedulers.

According to our previous results, using random numbers instead of the residual capacity of the nodes showed better performance in the network lifetime; therefore, we are going to use this approach for our next evaluation. We now assess our ECKN algorithm, comparing its performance to CKN and with a solution using no sleep scheduler. Figure 17a shows the network lifetime using the different sleep schedulers. As can be seen, CKN using k=1 presents the best performance, followed by our ECKN algorithm, CKN using k=2, CKN using k=3, and the approach with no sleep scheduler presents the worst performance. The results are explained because CKN using k=1 has the highest number of nodes sleeping per epoch, once each node is required to have only one awake neighbor. ECKN is the next because most of the network nodes have k=1, only those closer to the sink have a larger value for *k*. The performance of CKN using k=2 and k=3 underperforms because they must have at least two and three awake neighbors in each epoch, respectively. No sleep scheduler presents the worst performance because no node goes to sleep; therefore, the whole network is awake, decreasing the network lifetime. Figure 17b shows the number of alive nodes at the end of the simulations. Following the network lifetime, CKN using k=1 has the best performance, followed by ECKN, CKN using k=2, CKN using k=3, and the approach with no sleep scheduler. Lastly, we evaluate the delivery ratio of the proposed sleep scheduler. As can be seen in Figure 18, the approach with no sleep scheduler presents the best delivery ratio; this is due to the fact that no node is sleeping. Therefore, the best path and shortest path can be used, lowering the probability of dropping a packet. CKN using k=3 shows the second-best delivery ratio, following the same idea; when we use k=3, each node must have at least 3 awake neighbors. This increases the probability of finding the best node to forward the packet. Following the same idea, CKN using k=2 presents a worse delivery ratio than CKN using k=3 and CKN using k=1 has the worst delivery ratio. ECKN presents a similar performance to CKN using k=3, this is due to the fact that nodes closer to the sink have larger values for *k*, increasing the probability of reaching the sink. Therefore, due to the different values of *k* according to the distance to the sink, ECKN performs as well as CKN using k=1 in terms of the network lifetime, while it maintains a good delivery ratio, as good as the one presented by CKN using k=3.

## 7. Conclusions

This paper introduces a new algorithm called ECKN for wireless sensor networks that combines localization estimation, packet routing, and sleep scheduling. ECKN uses a mobile sink to estimate sensor node positions through trilateration, and proposes algorithms for accepting or rejecting position packets from the mobile sink. The results indicate that ECKN enhances the network lifetime while maintaining the packet delivery ratio. In addition to the ECKN algorithm, the paper presents an algorithm for forwarding event packets from sensor nodes to the sink based on the distance and residual energy of neighboring nodes. It also introduces bridges as a way to reduce the number of intermediate nodes between the source sensor node and the sink, thus shortening paths.

The proposed sleep scheduling algorithm is based on CKN, which ensures network connectivity with each node having at least k awake neighbors. The proposed algorithm assigns different k values based on the node’s distance from the sink, with nodes closer to the sink having a larger k value to handle higher network traffic and improve the network lifetime.

The paper proposes a localization algorithm that utilizes a mobile sink for trilateration-based sensor node position estimation. Additionally, it presents algorithms for finding reference points and accepting/rejecting position packets. ECKN with bridges outperforms other algorithms in terms of the packet delivery ratio and number of hops. The sleep scheduling algorithm assigns varying k values based on the distance to the sink, similar to CKN, for the network lifetime and delivery ratio.

Based on simulation experiments, the proposed ECKN with bridges outperforms ECKN without bridges and GGF. This superiority is attributed to the creation of bridges by ECKN, which reduces the distance between the source node and the sink, thereby increasing the likelihood of successful packet delivery. ECKN without bridges lags behind GGF because ECKN takes into account not only the node positions but also their remaining energy. This additional consideration can sometimes result in longer paths for packets, thereby increasing the chances of packet drops. The success of packet delivery depends on the number of hops taken by the event packet from the source to the sink node. ECKN with bridges achieves the lowest average number of hops due to the creation of bridges, which effectively shortens the path between the source and sink nodes compared to GGF.

In terms of network overhead, GGF and ECKN without bridges exhibit similar behavior, while ECKN with bridges incurs higher overhead. This discrepancy can be attributed to the larger time-to-live (TTL) used by ECKN with bridges during bridge creation. Unlike GGF and ECKN without bridges, which maintain the same TTL for every position packet from the sink, ECKN with bridges adjusts the TTL based on the possibility of shortening the path between an area and the sink’s position. When a positive adjustment is made, increasing the TTL of the position packet allows it to reach a more distant region. Consequently, the position packet is resent more times, leading to increased network overhead.

Regarding the network lifetime, ECKN demonstrates similar performance to CKN using k = 3. This similarity arises from the fact that nodes closer to the sink have larger values for k, which enhances the probability of reaching the sink. Therefore, due to the varying values of k based on the distance to the sink, ECKN performs as well as CKN using k = 1 in terms of the network lifetime while maintaining a good delivery ratio comparable to CKN using k = 3.

In future work, we plan to investigate the impact of a different number of sinks and sink mobility in the position estimation of the nodes and packet routing. We also plan to work on a failure recovery algorithm in the case where a packet is dropped in the packet routing process. Regarding the limitations of this work, node density plays an important role in the proposed approach as well as for most WSN solutions based on constrained sensor nodes. A carry-and-forward approach would have to be investigated to accommodate for low node density areas. In addition, the proposed approach relies on a mobile sink node, which can be a bottleneck and a single point of failure in the system. Adding more sink nodes may mitigate this issue, however, it will introduce more overhead. This has to be carefully investigated in future works. 

## Figures and Tables

**Table 1:** Sink position packet.

Field	Type	Description
sqnNum	integer	Sequence number of the packet, the nodes are going to update the position of the sink only if the sqnNum is bigger than the previous received.
posX	double	Position of the sink in the X-axis.
posY	double	Position of the sink in the Y-axis.
ttl	integer	The nodes are going to forward the sink position packet to its neighborhood until ttl is 0.

**Table 2:** Sensor node position packet.

Field	Type	Description
address	string	The address of the sensor node.
battery	double	The power remains in the battery of the sensor node.
position X	double	Position of the sensor node in the X-axis.
position Y	double	Position of the sensor node in the Y-axis.
accurate	Boolean	Whether or not the position of the sensor node is accurate.

**Table 3:** Evaluation parameters.

Parameter	Value
Network Size	1000 × 1000 m${}^{2}$
Number of sensor nodes	50, 75, 100, 125, and 150
Number of sinks	1
Sink start position	(500, 500)
Sink avg speed	10 m/s
Sink mobility model	Random waypoint
Sink beacon frequency	5 s
Transmission range	185 m
Position distance threshold	50 m

## References

[1] Akyildiz I.F., Su W., Sankarasubramaniam Y., Cayirci E. (2002). Wireless sensor networks: A survey. Comput. Netw..

[2] Bhuiyan M.Z.A., Wang G., Vasilakos A.V. (2015). Local area prediction-based mobile target tracking in wireless sensor networks. IEEE Trans. Comput..

[3] Zhu C., Yang L.T., Shu L., Leung V.C., Rodrigues J.J., Wang L. (2014). Sleep scheduling for geographic routing in duty-cycled mobile sensor networks. IEEE Trans. Ind. Electron..

[4] Frey H., Stojmenovic I. On delivery guarantees of face and combined greedy-face routing in ad hoc and sensor networks. Proceedings of the 12th Annual International Conference on Mobile Computing and Networking.

[5] Falcon R., Liu H., Nayak A., Stojmenovic I. Controlled straight mobility and energy-aware routing in robotic wireless sensor networks. Proceedings of the 2012 IEEE 8th International Conference on Distributed Computing in Sensor Systems (DCOSS).

[6] Li M., Li Z., Vasilakos A.V. (2013). A survey on topology control in wireless sensor networks: Taxonomy, comparative study, and open issues. Proc. IEEE.

[7] Hofmann-Wellenhof B., Lichtenegger H., Collins J. (2012). Global Positioning System: Theory and Practice.

[8] Bulusu N., Heidemann J., Estrin D. (2000). GPS-less low-cost outdoor localization for very small devices. IEEE Pers. Commun..

[9] Williams S.D., Bock Y., Fang P., Jamason P., Nikolaidis R.M., Prawirodirdjo L., Miller M., Johnson D.J. (2004). Error analysis of continuous GPS position time series. J. Geophys. Res. Solid Earth.

[10] Tolani M., Bajpai M., Balodi A., Sunny A., Singh K.R. (2023). Energy-Efficient Duty-Cycle Hybrid Medium Access Control Protocol for Wireless Sensor Network. Wirel. Pers. Commun..

[11] Ren Q., Yao G. (2022). An Opportunistic Routing for Energy-Harvesting Wireless Sensor Networks With Dynamic Transmission Power and Duty Cycle. IEEE Access.

[12] Luomala J., Hakala I. (2022). Adaptive range-based localization algorithm based on trilateration and reference node selection for outdoor wireless sensor networks. Comput. Netw..

[13] Pandey S., Varma S. (2016). A Range Based Localization System in Multihop Wireless Sensor Networks: A Distributed Cooperative Approach. Wirel. Pers. Commun..

[14] Mani R., Rios-Navarro A., Sevillano-Ramos J.L., Liouane N. (2023). Improved 3D localization algorithm for large scale wireless sensor networks. Wirel. Netw..

[15] Chen C.C., Chang C.Y., Li Y.N. (2013). Range-free localization scheme in wireless sensor networks based on bilateration. Int. J. Distrib. Sens. Netw..

[16] Karp B., Kung H.T. GPSR: Greedy perimeter stateless routing for wireless networks. Proceedings of the 6th Annual International Conference on Mobile Computing and Networking.

[17] Toussaint G.T. (1980). The relative neighbourhood graph of a finite planar set. Pattern Recognit..

[18] Gabriel K.R., Sokal R.R. (1969). A new statistical approach to geographic variation analysis. Syst. Biol..

[19] Kumar P., Hariharan R. (2022). Improved trustworthy, speed, and energy-efficient GPSR routing algorithm in large-scale WSN. Meas. Sens..

[20] Bose P., Morin P., Stojmenović I., Urrutia J. (2001). Routing with guaranteed delivery in ad hoc wireless networks. Wirel. Netw..

[21] Mineno H., Soga K., Takenaka T., Terashima Y., Mizuno T. (2011). Integrated protocol for optimized link state routing and localization: OLSR-L. Simul. Model. Pract. Theory.

[22] Clausen T., Jacquet P. (2003). Optimized Link State Routing Protocol (OLSR).

[23] Takenaka T., Mineno H., Tokunaga Y., Miyauchi N., Mizuno T. (2007). Performance analysis of optimized link state routing-based localization. J. Inf. Process. Soc. Jpn. (JIP).

[24] Oliva G., Panzieri S., Pascucci F., Setola R. (2013). Simultaneous localization and routing in sensor networks using shadow edges. IFAC Proc. Vol..

[25] Jiang M. (1999). Cluster Based Routing Protocol (CBRP).

[26] Kirci P., Chaouchi H. (2016). Recursive and ad hoc routing based localization in wireless sensor networks. Comput. Stand. Interfaces.

[27] Cota-Ruiz J., Rivas-Perea P., Sifuentes E., Gonzalez-Landaeta R. (2016). A Recursive Shortest Path Routing Algorithm With Application for Wireless Sensor Network Localization. IEEE Sens. J..

[28] Chen B., Jamieson K., Balakrishnan H., Morris R. (2002). Span: An energy-efficient coordination algorithm for topology maintenance in ad hoc wireless networks. Wirel. Netw..

[29] Cerpa A., Estrin D. (2004). ASCENT: Adaptive self-configuring sensor networks topologies. IEEE Trans. Mob. Comput..

[30] Godfrey P., Ratajczak D. Naps: Scalable, robust topology management in wireless ad hoc networks. Proceedings of the 3rd International Symposium on Information Processing in Sensor Networks.

[31] Schurgers C., Tsiatsis V., Srivastava M.B. STEM: Topology management for energy efficient sensor networks. Proceedings of the Aerospace Conference Proceedings.

[32] Yang X., Vaidya N.H. A wakeup scheme for sensor networks: Achieving balance between energy saving and end-to-end delay. Proceedings of the Real-Time and Embedded Technology and Applications Symposium.

[33] Nath S., Gibbons P.B. Communicating via fireflies: Geographic routing on duty-cycled sensors. Proceedings of the Information Processing in Sensor Networks.

[34] Yuan Z., Wang L., Shu L., Hara T., Qin Z. A balanced energy consumption sleep scheduling algorithm in wireless sensor networks. Proceedings of the Wireless Communications and Mobile Computing Conference (IWCMC).

[35] Reich J., Misra V., Rubenstein D., Zussman G. (2012). Connectivity maintenance in mobile wireless networks via constrained mobility. IEEE J. Sel. Areas Commun..

[36] Erceg V., Greenstein L.J., Tjandra S.Y., Parkoff S.R., Gupta A., Kulic B., Julius A.A., Bianchi R. (1999). An empirically based path loss model for wireless channels in suburban environments. IEEE J. Sel. Areas Commun..

[37] Forbes C., Evans M., Hastings N., Peacock B. (2011). Student’s t Distribution. Statistical Distributions.

